# History of Tuberculosis, with a Special Consideration of Tuberculosis in Cattle

**Published:** 1884-01

**Authors:** Albert Johne

**Affiliations:** Professor at the Royal Veterinary School, Dresden, Germany


### ART. VI.—HISTORY OF TUBERCULOSIS, WITH A SPECIAL CONSIDERATION OF TUBERCULOSIS IN CATTLE.

BY DR. ALBERT JOHNE,

*Professor at the Royal Veterinary School, Dresden, Germany.* *Translated from the Deutsche Zeitschrift für Thiermedicine und vergleichende Pathologie.—Vol. IX., No.* 1, 2.

### INTRODUCTION.

A review of the work accomplished within the past century the field of general pathology developes the fact that probably few diseases have enjoyed a greater amount of discussion by the most prominent thinkers than tuberculosis. The purpose, however, to which all these labors were directed was a noble one. It was not only to break the power of one of the most dreadful scourges of mankind, but also to investigate its ætiological connection with bovine tuberculosis, and in this way to determine the nature of a disease, the eminent importance of which for public health as well, as for stock-raising can no longer be denied.

This noble end has not yet been fully attained. Many questions of the greatest importance remain unsolved. But since the publication of Villemin's (1865) able works on this subject, it has become gradually established that tuberculosis in man and animals is not only one of the most frequent, but on account of its incurability one of the most dreaded diseases, or, as Cohnhein and Koch pronounce it, the product of our social and economical conditions and it must be looked upon as a transmissible infectious disease.

And even more! The genial investigator on mycology, Koch, of Berlin, has succeeded in proving positively, not only the parasitic nature of the tubercular virus in general, but also the presence of the bacillus found by him, in tubercles in man and animals, and in this way has shown the identity of both processes.

I do not think it is going too far to assert that the latest work of Koch has settled the question of the genesis of tuberculosis in its principal features, and that we may now discuss the question as to the consequences which must result from the infectious nature of tuberculosis.

The result of the endeavors of most eminent investigators for the past thirty years compels us to turn our attention most earnestly to the relation which exist between tuberculosis in animals and man.

The carelessness, which in many cases might be called reprehensible, with which agriculturalists have tolerated tuberculosis in their cattle, must be abolished, and the medical and veterinary professions will be compelled to assume a most decided position on the question of tuberculosis.

The history of tuberculosis has arrived at a point, the full importance of which can as yet be hardly appreciated. This state of things does not come upon us unexpectedly, however, but has been anticipated and is but a necessary sequence to those who have followed the labors in this field of research attentively.

It might be a grateful task at the present time to look back upon the development of our knowledge on the subject of tuberculosis and to give our special attention to bovine tuberculosis, especially as the latter can now be said to be celebrating its centennial anniversary.

In 1772 Kreisphysicus Heim rendered an opinion to the chief sanitary commmission of Berlin, on the nature of beef from cattle affected with Perlsucht, and this opinion marks the period at which bovine tuberculosis lost all importance in human and veterinary medicine. On the other hand the discovery made by Koch in 1882, forms the keystone of a series of very interesting investigations on the infectiousness and unity of tuberculous processes in man and animals; and with this discovery bovine tuberculosis became again a subject of great hygienic importance.

### Part I.—History of Tuberculosis.

*Period before Villemin.*—The doctrine of tuberculosis, as at present considered, is comparatively young, as it dates back only as far as the last ten years of the eighteenth century. Before this era the term "tuberculosis" was used in a descriptive sense only for the designation of node-like formations of every description.

One class, known as miliary tubercles, surrounded by a vascular rête, but having no central vessel and not known to grow over a certain size, was not known to the ancients or even to investigators in the middle ages. The larger nodes resulting from the confluence of these minute miliary tubercles have been, up to the latter part of the eighteenth century, only known as simple abscesses without any specific characteristics.

According to ancient authors on medicine, Hippocrates, Celsus, Aretaeus, Galenus, etc., phthisis consisted in nothing else than the formation of abscesses or ulcers in the lungs, in spite of the fact that the first named author had written a very able description of pulmonary consumption. The term "tubercle," as meaning a solid node, is first met with in the works of those investigators who, from the recognition of anatomy as an independent science, were enable to make more frequent examinations of the dead body. In these researches there were very often found within the lungs different nodular formations, which were sometimes called tubercles, at others scirrhus, struma or steatoma, without any efforts being made to bring them into any ætiological connection with pulmonary consumption. The first who asserted an ætiological relation to exist between the nodes in the lungs and phthisis was Sylvius, (Vol. II. p. 622) who announced, in 1695, that he was convinced that phthisis, sometimes resulted from smaller or larger nodes in the lungs, and in part from catarrh and pneumonia, that both processes finally lead to abscesses and formation of cavities. Sylvius was also the first to assert that the small nodes in the lungs grew from very minute lymphatic glands, which were invisible to the eye, but, which, under certain conditions in so-called scrofulous constitutions, grew, became visible, and after attaining a certain size, changed into abscesses.

Miliary tubercles were, without doubt, described by Manget, who (in re-writing the great work of Bennet, published in 1700, one of the first on the subject of Pathologic Anatomy,) states that in the dissection of the body of a person that had died of phthisis that he discovered nodes in the lungs, the liver, spleen, kidneys, and intestines of the size of a millet seed—semen milii. He described the cheesy degeneration of the nodes, but considered them to be minute lymph glands. Morton, Sydenham, Leigh, Mend, van Swieten and Morgagni had almost the same ideas.

Stark, whose work was published 1785, fifteen years after the author had died, was the first to attribute greater importance to miliary tubercles, of which he gave a very distinct description. Reid fully adopting the views of Stark, (as did also Cullen) went one step further. He was the first (1785) to consider miliary tubercles as independent neoplastic growths having nothing to do with enlarged lymphatic glands viz. scrofulosis; he denied also the existence of small lymphatic glands within the parenchyma of the lungs. This opinion remained unsupported for a time, his cotemporaries and successors, Cullen, Kortum, Hufeland, Baume and others supporting the idea that the nodes in the lungs were glands, viz. scrofulæ, that owed their existence to the influence of a so-called scrofulous acid that had been secreted by pre-existing lymphatic glands in the lungs.

Baillie was the only one to consider the discovery of Stark to a further degree. He not only declared himself as decidedly opposed to the co-ordination of tuberculosis and scrofulous nodes, but went one step further in proving that the larger nodes in the lungs were only the result of the confluence of small miliary nodes. (His work on pathologic anatomy was translated into German and published 1794.) He distinguished these nodes decidedly from other formations in the lungs, and classed the latter as "scrofulous matter," or "scrofulous infiltrations." Portal, who lived at the same time, agreed in general with these views; he considered the formations that Baille had considered as "scrofulous" as "tuberculous." This was a mistake which received endorsement later by Laënnec.

The real founder of the doctrine of Tuberculosis was Bayle. He introduced, beginning of the nineteenth century, into the vocabulary of science the term miliary tubercles for the small nodes and described exactly all the phases of their development as well as the part which they played in forming larger tubercles. He accurately recognized the genetical as well as the clinical relation existing between the tuberculous processes in the different organs of the same organism. He considered tuberculosis to be a general disease, the origin of which was in a tuberculous diathesis.

This doctrine was adopted by Laënnec in his work on diseases of the heart and lungs (published 1819). This investigator described the history of the development of tubercles in a most classical manner, but he went, unfortunately, one step farther than Bayle in classing each and every cheesy infiltration wherever found, even if the same was not sharply defined as "tuberculous." In this way he made cheesy degeneration, the principal characteristic of tuberculosis, a view of the subject which was closely followed by Rokitansky, Reinhard and others, even if there was but a simple retrogressive metamorphosis or a common inflammation existing.

To Virchow is due the credit of bringing order out of this chaos of ideas. He proved, that cheese faction was not only connected with certain pathological processes and limited to particular tissues, but that in all possible formations of an inflammatory or hyperplastic nature, under certain conditions of nutrition, the process of cheese faction could occur. As tuberculosis, were to be classified only those processes which resulted from submiliaric nodes, devoid of vessels, resembling lymph-follicles and composed of round, lymph-like cells, growing only to the size of a millet seed, and having arrived at this stage undergoing the process of cheesy degeneration.

From these genuine tuberculous processes, Virchow separated all those cheesefactions that appear under certain hyperplastic or inflammatory conditions, as for instance in scrofulous tumors of the lymph glands, in caseous pneumonia, or even in the common exudates and in tumors of different kinds. Not cheesy degeneration as such, but its springing forth from submiliar nodes is the characteristic of tuberculosis. With this assertion he assumed a decidedly anatomical point of view and considered the tubercle to be a lymphoid formation, developing from the elements of true connective tissue, and due to specific irritation.

Virchow, at the same time, denied the existence of genuine tubercles in animals (p. 716) and attributed their occurrence to a general or local diathesis, which consisted in a specific predisposition of the tissue, in consequence of which not only specific irritants, but also causes of a general nature as colds could lead to the generation of the disease. This predisposition which may be caused by insufficient nourishment, inherited or innate, involves at the same time a degeneration of the blood, in consequence of which the parts of the body requiring nourishment receive insufficient material, so that they can no longer continue in a normal condition.

Virchow also recognized the infectious property of tuberculosis, not only when in a condition of cheesy degeneration, but when in one of active proliferation, the infectious element being dispersed over the organism either by means of the blood vessels or lymphatics metastasis, or by the extension of the diseases by the development of new tubercles in the immediate vicinity of older ones, the latter infecting the tissues in immediate opposition with them. In this way Virchow plainly distinguished between scrofulosis and tuberculosis. He characterized the latter as an inflammatory or hyperplastic process, the products of which undergo retrogressive changes at an early period of life. Now if the final results of both tuberculosis and scrofulosis be cheesy metamorphosis of pre-existing elements still we must distinguish between the fundamental processes of scrofula and tuberculosis, as they are entirely different.

Nearly at the same time (1857) Buhl advanced a new theory on the subject of tuberculosis, which has only been partially adopted by Virchow. This theory has many of the most competent investigators amongst its advocates. Buhl went a little farther than Virchow. According to his theory tuberculosis is a specific resorbent and infectious disease, caused by a particular poison, called "tuberculous virus," which is formed in cheesy matter of every description, contrary to the assertion of Virchow, who thinks that it can be formed only in local and primary tubercles. If the virus by way of absorption be brought into the blood it can generate miliary tubercles in all predisposed organs, which, in their turn, rendering cheesy degeneration, can become a source of constant self-infection to the organism. This theory of the infectiousness of cheesy material by means of a specific virus generated in it somewhat resembles the views of Dittrich, who considered that tuberculosis could be caused by the absorption of the products of all sorts of necrobiotic processes by the circulation.

ARMY MEDICAL LIBRARY
[illegible]NGTON, D. C. U. S. A.

This theory of Buhl became the herald of a new doctrine, which gained the victory in a contest that lasted nearly twenty years, the object of it being to decide the true nature of tuberculosis.

Previous to Buhl various experiments had been made with regard to the transmissibility of the infectious principle of human tuberculosis; of these may be mentioned those of Kortum (1789), Hebréard (1802), Salmade (1805), Lepelletier (1830), Goodlad and Deygallieres (1829), Laënnec, Erdt (1834), and others. After Klenke had reported (1843), that he had succeeded in producing general tuberculosis in the liver and lungs by the injection of tuberculous matter, taken from tuberculous infiltrations, into the jugular of a rabbit, Villemin declared (1864), tuberculosis to be a specific infectious disease. He declared tuberculosis to be a disease, independent of other internal or external circumstances, that can only be caused by the incorporation of tuberculous substances into the organism, and which can be transferred from animal to animal or from man to animal by means of vaccination.

It is seldom that an assertion, so decidedly pronounced, has given greater impulse to scientific labors in the field of experimental pathology than the new doctrine of Villemin. The most prominent men of science have called into activity all their powers as well as sagacity, either to establish and prove, or to contradict the new doctrine.

It is self-evident that all these investigations have been of great influence upon the knowledge of bovine tuberculosis, and in this way have received the consideration which is due to the important subject of public hygiene. From this point of view it seems justifiable to the history of bovine tuberculosis back to the time when Villemin first called the attention of investigators to the same and in this way closely connect the history of human and bovine tuberculosis.

The principal forms of bovine tuberculosis, of which we shall speak particularly, those of the lungs and those of the pleura and peritoneum have not been regarded as one and the same disease. Each of them has its own history.

The oldest form is in ancient works on veterinary medicine sometimes termed consumption, phthisis pulmonum ulcerosa, and is the same as tuberculosis in man (phthisis pulmonum).

As in pulmonary consumption in man, the point in question here is also a confluence of the different processes in the lungs, between which a gradual separation has only been accomplished at a very recent date.

In regard to the ancient ideas it will be sufficient to state that the latter, in their cardinal points, coincides with the history of consumption in the lungs of man. As all ancient authors on this subject, up to a very recent date, by the term "consumption of the lungs" in man, simply expressed the idea of ulceration or suppuration of the lungs, using this collective term for the most variable conditions, as we also find veterinary authors speaking of phthisis pulmonum in the same sense. This might be easily explained, if we consider that the first beginnings of independent veterinary medicine can not be dated back farther than to the middle of the eighteenth century, and that the science of veterinary medicine in its present shape must be considered a product of the present day. What has been accomplished in the scientific study of diseases in animals is, almost without exception, the work of men who devoted themselves especially to the investigation and treatment of the diseases of man. There is even reason to believe that many of the facts related by ancient Greek and Roman authors in connection with consumption in the lungs of men are partly derived from observations made in the dissection of dead animals, as the fact is well known that the obtaining of human bodies for the purpose of anatomical dissection at that time was surrounded by great difficulties. One of the oldest authors that mentions consumption of the lungs in cattle, is Columella. (40 a. c.) He speaks also of an ulceration of the lungs, (exulceratio pulmonum). That the ancient jewish legislators were acquainted with bovine tuberculosis, viz., consumption of the lungs, appears from the facts reported by Waldenburg (p. 24). The defects reported in the "Mischna," which was edited towards the end of the eleventh century, as holes (perforating ulcers) and other defects of the lungs, windpipe, stomach, heart, etc., may, with a degree of certainty, be considered as relating to diseases of the lungs. It also is the case with the facts in the "Gemara, edited (500 A. D. In the latter work we find obstruction of the lungs by ulceric matter mentioned, and also abscesses filled with pus in the lungs. According to Waldenburg the hard and heavy tumefactions, which are mentioned by the French author, Raschi, (died 1105) as having been found in the lungs of cattle, may be considered to refer to bovine tuberculosis.

All the later authors on the diseases of animals, until the nineteenth century, had the same idea with regard to consumption in the lungs. They all speak of nodes and schirrous tumors in the lungs that transform into ulcers and end in the destruction of the tissue of the lungs—Willburg, Seeger, Erxleben, Pilger, Kitt and others. The investigations made toward the end of the eighteenth century by Chabert and Hazard in reference to a lung-disease among milch cows which had been raging for about twenty years in Paris and vicinity, demonstrate beyond all doubt that both of them considered phthisis of the lungs in the old sense, and that both had no idea of bovine tuberculosis in the present scientific meaning of this term. Even when Stark (1785), Reid (1785), and Baillie (1794) had discovered and very distinctly described the miliar tubercle, and the latter had pointed out its essential connection in the formation of larger tubercles, aud Bayle had finally given the doctrine of tuberculosis a solid foundation, even then the old ideas of consumption remained in veterinary medicine, as shown by the works of Hofacker, Tscheulin, Ribbe and others. In no case have any of the above mentioned authors ever used the term "tubercle" in other than a descriptive sense.

Dupuy seems to have been the first who applied this term in 1817) in the doctrinary sense in Veterinary medicine, according to the theory of his great countryman, Baillie. He began by describing tuberculosis as it appeared in the lungs of diseased horses in glanders and farcy, the latter, however, according to later investigations, are not to be identified with tuberculosis. In this connection he treated phthisis of monkeys, tuberculosis of the lungs and membranes of cattle, but fell into the mistake of bringing these into connection with the formation of all possible kinds of Hydatids, a mistake that had crept into medicine as early as Hippocrates and that had been, according to Dupuy, still adhered to by Baron and Ruhu in connection with human tubercles. A treatise on tuberculosis of the lungs, published soon after Dupuy (1818), by Veith, adheres completely to the old standpoint. Tubercles are only mentioned in connection with the disease of glanders. The tuberculosis nature of the serous membranes was known neither to him nor Dietrichs.

Gurlt admitted (1831) the tuberculous nature of consumption of the lungs. He gives a very accurate description of the larger tubercles and the changes they undergo. He considered consumption of the lungs, which was said to occur in cows, as tnbercular consumption (phthisis tuberculosis). These nodes were said to be present in healthy tissue and to undergo the same processes which Bayle described as characteristic of tuberculosis. All softenened cheesy nodes in the lungs were, without exception, tubercles, as it was stated that suppuration very seldom occurred in the lungs. Gurlt took the standpoint of Laënnec, a conception which we also find in Spinola 1839. Rychner and Funke agree fully with the views of Gurlt, while Hering has made endeavors to distinguish between the pathologic processes that lead to a consumption in the lungs. He pronounces the disease that is most frequently met with in milk-yielding cattle as obstinate and of long duration without fever hardening or suppuration of the substance of the lungs, as partly the consequence of acute diseases of the lungs, chronic catarrh, etc., partly as the result of the formation of tubercles.

The tubercles that have been formed by exudations from the blood vessels have in their first stages a red areola, and later turn into a gray-yellowish hue (nodular lobular pneumonies). But Hering was right in maintaining that larger neoplastic formations were due in most cases to inflammation of the lungs, but sometimes could have sprung up from the confluence of several smaller, softened tubercles.

Up to this time of all physicians that had studied the pathologic anatomy of the human body only Gluge paid special attention to tuberculosis of animals. According to his investigation this disease appears in all the organs in cattle, particularly as tuberculosis of the peritoneum and the lungs. A similar result was attained by Forster. He did not suppose bovine tuberculosis to be identical with tuberculosis in man, but he pointed out that tubercles appeared partly in the serous membranes, partly in the lungs, but that in the lungs they were met with in two different forms: First, in the shape of gray tubercles in the interstitial tissues of the lungs, being partly miliary, partly larger, assuming later a cheesy condition and by confluence forming tubercles of different size, filled with paste-like detritus. Second, in the shape of lobular tuberculosis, cheesy pneumonies. He also mentions the bronchictatic processes that are frequently connected with both forms which have been more particularly described by Bruckmueller and Siedangrotzky.

The first works of the last mentioned investigator on this subject had already been published before the works of Foster. Virchow at the meeting of the Physical Medical Society in Wurzburg, May 12th, 1855, entered more specially into the subject of bovine tuberculosis. But he was not justified in transferring the term "French disease," or "perlsucht," that had heretofore been limited to the node-like formations in the serous membranes to the neoplasms in the interior of the organs (as lungs, liver bronchial-glands, uterus, tubes, etc.,) which had, up to that time, been called tubercles. This was an error that had already been committed by Förster and afterwards by most of the authors in medicine. Virchow acknowledged the great resemblance of the new formations in regard to their development and to their relation to local and constitutional disease to tuberculosis, but he nevertheless grouped them with "lympho sarcomen" in the human body. As is well known, this author has adhered to this idea up to the present day, and not only has defended the same in all his later works on this subject, but declared the existence of the genuine (human) tubercles in the lungs of animals, especially of cattle to be very doubtful.

The history of this form of tuberculosis in the serous membranes of animals, especially in cattle, or the "perlsucht" proper is comparatively short, but the many changes of views as to its real nature, are the more interesting. This is shown by the great number of terms applied to the same which it seems have been invented only in default of a clear and distinct knowledge of its nature. It has been termed franzosenkrankheit, venerie, lustseuche, geilsucht, geile seuche, unreinigkeit, monatsreiterei, hirsesucht, fraubenkraukheit, meerlinsigkeit, rindshamnen, kramen, graunigt, finnig, or krättigsein zäpfigkeit, drüsenkrankheit, sarkomdyskrasie, perlsucht, fibrous tuberculosis, primary tuberculosis of the serous membrane, cattle tuberculosis, morbus gallicus boum, nymphomania, satyriasis, parresyge, cachexia boum sarcomatosa, sarcomatosis infectiosa, sarco-tuberculosis boum infectiosa, tuberculosis serosa boum, tuberculosis glandularis boum, cachexia tuberculosis boum, cachexia vaccarum tuberculosa, tuberculosis pleuralis, tuberculosis boum fibromatosa, margarosis, la pommelière, phthisie crétosée, phthisie calcairée, etc., etc.

It is singular that neither the ancient authors on veterinary medicine, nor the regulations for killing cattle with the ancient jews, mention the very many characteristic nodular neoplasms on the serous membranes of the abdomen and the chest, which in many characteristic points resemble perlsucht. Neither has this been done in the decrees, issued by the clerical and secular authorities relating to the marketability of the meat of diseased animals as early as the middle of the eighth century. According to Graumann, to whom we are indebted for the first detailed description of perlsucht, which was, until then, generally known as "French disease," some decrees were issued in 1680, among them is mentioned unclean cattle. Florini, who does not seem to have known of the above mentioned decree, applied the term "French disease" in 1702 for the first time. Furstenan (1747) describes this disease in quite an extended manner. He believed it to be a venereal process. This latter assertion was denounced by Zink (1764). Krunitz states (1778) that the disease was only known in name. Dr. Ruhlîng, in Northeim, on the other hand, observed (1774,) the endemic prevalence of the "French disease" among cattle and mentions (1796) that it was not only a hereditary disease, but also the fact that of a flock of 100 head three or four head had annually fallen victims to this disease.

The reasons for the decrees prohibiting the use of the meat from "French" cattle, as well as the origin of the word "French disease," may, according to Graumann be found in the following: "At the time when these decress were issued people lived under the full impression of the terror that had been caused by the epidemic spread of the venereal disease among men (which, according to Baumler, towards the end of the fifteenth century, had been recognized as

"morbus gallicus," or "franzosen krankheit". Of this pestilential plague neither the origin nor the means by which it spread so rapidly had become known, and in looking for an explanation for both, Helmont had arrived at the idea that "sodomy" was the much dreaded cause. From this Graumann concluded, that if criminal cohabitation could create a venereal pest in man, it could be taken for granted that an animal, abused in the same way, would be infested by it. If by examination of the cadaver of a cow that had been killed for this cause were found such unuatural formations, the real nature of which was not known at that time, people were easily induced to look upon them as signs of infection or proofs of the existence of a venereal disease. If another cow was found with similar formations, it was thought that the animal had been secretly abused and bore the proofs of this unclean, disastrous cohabitation in her body. Such animals were declared uneatable, and according to the custom of the time were condemned to be burned.

This assumption of Helmont has exerted no inconsiderable inflnence. The belief that the use of meat impregnated with venereal poison was hurtful, had, in course of time, taken root, and the governments, after having once had their attention called to it, prohibited the use of such meat altogether. Stubbornness and superstition also did their work, and conditions soon developed, of which at the present day we can scarcely form any conception. Graumann says on this subject:

> If an animal, in the interior of its body, had certain grape shaped excrescences that hang on the interior membranes, it is believed that the animal has the "franzosen" and is infected. Nothing more is required than the presence of these tumors, and no further examination of the dead animal is deemed necessary, for as soon as the butcher sees these unnatural appendages he throws away the knife and does not touch the animal any more—it was to be given to the knocker. In some localities, where superstition and prejudice is still more powerful, the butcher has to pay a fine of one Reichsthaler before he is allowed to butcher animals again. Not until such fine was paid would any honest "Zunftmeister" handle one of the polluted tools."

Whether the animal was fat or lean no distinction was made, they must go to the knocker, while all other diseases were not regarded as detrimental to the legal sale of the meat. After Zink had called attention to the harmless nature of the meat of "perlsuchtig" cattle, it was Graumann again, who, by order of the government of Necklenburg Schwerin, made open war against these unsuitable conditions. He declared the "franzosen," the location and shape of which he very accurately describes, as a kind of "hiclatides" (Echinococcen) that had probably assumed a peculiar appearance. This opinion has been repeatedly corroborated. (See Duburs, Hofacker, Hayne, Viborg, D'Arboval and others.) Compare also Rayer and Leonhardt, 1880, on the accidental occurrence of echinococci tuberculosis in cattle. He says the reason of the so-called "French disease" is an abundance of fluids which can not be kept in their proper channels, but burst them, run out and form the tumors. He denounces, together with Kersting, who, at the same time, by order of the government of Necklenburg Strelitz, rendered an opinion on the same disease, the peculiarity of its being inherited and claims their origin to be due to luxuriant pasturage or to too damp pasture, and finally ventured the assertion that it was no real and proper disease at all.

In a similar sense Kreisphysicus Heim, in his report to the "Ober-Sanitats Collegium," Nov. 26th, 1792, declared against its syphilitic nature and in favor of the marketability of the meat of "perlsuchtige" cattle. But nevertheless there appeared in the following a regulation to decide whether a certain animal had been infected by the "French disease." This "regulation" had sprung up under the full impression of the truth of the views, that had been contradicted by Graumann and Heim, and prohibited entirely the use of meat of this kind. The description given in the above mentioned document does not apply to any of the diseases in cattle and can only be suitably taken as venery in man. According to the above mentioned regulation there will be sometimes found in completely sound and well-fed cattle, in some parts of the intestines, spongy, hardened, grape-like tumors, which are not to be classed as "franzosen," and after the removal of which the meat can be used without detriment to health; the presence of the last mentioned swellings should not be considered sufficient reason for giving the whole carcass to the knocker. It was not until the 27th day of July, 1785, that by decree of the "general directorium" prohibitary decrees on the subject were rescinded in Prussia. This measure was soon followed in Austria (July 11th, 1788), and later in all German States. It was, however, not so easy to uproot old prejudices and according to Viborg, the apathy against meat of this kind is said to have still existed in 1810, while in Norway continued until 1818.

With the act of repealing the prohibitory decrees, the "perlsucht" had been freed of its syphilitic character, but there was still very little knowledge as to the true character and the tuberculosis nature of this disease, and far less information regarding its identity with human tuberculosis. Tuberculosis of the lungs in cattle has been the subject of many investigations and scientific differences of opinion which culminated in the question: "Are perlsucht and tuberculosis in the lungs in cattle identical processes which are to be classed with tuberculosis?" The solving of this question was rendered difficult by the fact that the Germans frequently mixed up the abdominal form of this disease with nymphomanie, which often appeared connected with it, while French pathologists and their followers from the start give more prominence to the relations of perlsucht to tuberculosis and phthisis.

